# Gadd45a Is an RNA Binding Protein and Is Localized in Nuclear Speckles

**DOI:** 10.1371/journal.pone.0014500

**Published:** 2011-01-07

**Authors:** Yuliya A. Sytnikova, Andriy V. Kubarenko, Andrea Schäfer, Alexander N. R. Weber, Christof Niehrs

**Affiliations:** 1 Division of Molecular Embryology, DKFZ-ZMBH Alliance, Krebsforschungszentrum, Heidelberg, Germany; 2 Division of Toll-like Receptors and Cancer, Deutsches Krebsforschungszentrum, Heidelberg, Germany; 3 Institute of Molecular Biology (IMB), Mainz, Germany; New England Biolabs, Inc, United States of America

## Abstract

**Background:**

The Gadd45 proteins play important roles in growth control, maintenance of genomic stability, DNA repair, and apoptosis. Recently, Gadd45 proteins have also been implicated in epigenetic gene regulation by promoting active DNA demethylation. Gadd45 proteins have sequence homology with the L7Ae/L30e/S12e RNA binding superfamily of ribosomal proteins, which raises the question if they may interact directly with nucleic acids.

**Principal Findings:**

Here we show that Gadd45a binds RNA but not single- or double stranded DNA or methylated DNA *in vitro*. Sucrose density gradient centrifugation experiments demonstrate that Gadd45a is present in high molecular weight particles, which are RNase sensitive. Gadd45a displays RNase-sensitive colocalization in nuclear speckles with the RNA helicase p68 and the RNA binding protein SC35. A K45A point mutation defective in RNA binding was still active in DNA demethylation. This suggests that RNA binding is not absolutely essential for demethylation of an artificial substrate. A point mutation at G39 impared RNA binding, nuclear speckle localization and DNA demethylation, emphasizing its relevance for Gadd45a function.

**Significance:**

The results implicate RNA in Gadd45a function and suggest that Gadd45a is associated with a ribonucleoprotein particle.

## Introduction

The *Gadd45* genes are a family of stress response genes, which are involved in diverse processes, including cell growth, DNA repair, and apoptosis, and function as tumor- and autoimmune suppressors [1], [2]. Expression of these genes is induced by DNA-damage and genotoxic stress, including hyperosmotic stress and UV irradiation. The three *Gadd45* genes encode multifunctional, 18 kDa acidic proteins, which can homo- and heterodimerize and which are predominantly localized in the nucleus [3]. Gadd45 proteins interact with many effectors, including Cdc2/CyclinB1 [4], [5], PCNA [6], [7], p21 [8], nuclear hormone receptors [9], histones [10] and MEKK4 [11], [12], to mediate cell cycle arrest, differentiation or apoptosis.

More recently Gadd45 proteins have been implicated in epigenetic gene regulation, promoting active DNA demethylation via a DNA repair mechanism. Gadd45a binds to the repair endonuclease XPG and initiates excision repair at methylated CpG motifs in *Xenopus*, *Zebrafish*, and mammalian cells [13]–[18].

Gadd45 proteins exhibit sequence homology to the L7Ae/L30e/S12e superfamily [19]. Members of this family are diverse proteins from archea, eubacteria and eucaryota, including ribosomal proteins (S12, L30e), proteins that bind guiding RNA (L7Ae, 15.5 kD, fibrillarin), as well as components of ribonuclease P. Many of these proteins bind functionally diverse RNAs, including ribosomal RNA, snoRNA, snRNA and mRNA. Rather than binding to a specific consensus sequence, these proteins recognize a common structural motif – the kink turn, formed by both canonical Watson-Crick base pairing as well as and non-canonical interactions [20].

The fact that Gadd45 proteins belong to the L7Ae/L30e/S12e superfamily raises the question whether they may also bind RNA. Importantly, RNAs have been repeatedly implicated in active DNA demethylation although their history in this process is confusing [21]–[30]. Most recently ROS3 has been described as an essential mediator of DNA demethylation in *Arabidopsis*. ROS3 resides in nuclear speckle-like structures and binds small RNAs. It was suggested that these RNAs may guide the DNA demethylase towards their substrate [31].

Gadd45a has been shown to associate with chromatin [10], [13], [14], however, it is unknown whether it directly interacts with nucleic acids. Here we provide evidence that Gadd45a has RNA binding properties and possesses characteristics of a ribonucleoprotein particle (RNP).

## Methods

### Expression constructs and antibodies

For *Xenopus tropicalis* (xt) Gadd45a overexpression in human cells and *E.coli* we used constructs containing xtGadd45a ORF in vectors pRKW2 and pET28a as well as N-EGFP tagged xtGadd45a in pCS2 [13]. Point mutants of xtGadd45a, were obtained by circular PCR [32]. The following antibodies were used: anti-hGadd45a (H165), anti-p68 (H144), anti-Brg1 (N-15) (Santa Cruz), anti-hnRNP A1 and anti-histone H3 (Abcam), anti-GFP (Dianova), anti-SC35 (Novus Biologicals).

### Cell culture and transfections

HEK293T cells (ATCC CRL 11268) and RKO cells (ATCC CRL 2577) were grown at 37°C in 10% CO2 for 293T cells and 5% for RKO cells in Dulbecco's Modified Eagle's Medium (DMEM), 10% fetal calf serum, 2 mM L-Glutamine, 100 U/ml penicillin and 100 µg/ml streptomycin. Transient DNA transfections were carried out using FuGENE6 (Roche), TurboFect™ (Fermentas) in case of HEK293T, and for RKO cells a combination of Lipofectamine and Plus reagents (Invitrogen) was used following the manufacturer's instructions.

### Immunofluorescence microscopy

Cell detergent extraction and RNase treatment was essentially as described [33]. For detection of overexpressed Gadd45a (wild type and G39A mutant) RKO cells were transfected with pCS2-EGFP-Gadd45a. 24 h after transfection cells were extracted with 0.05% Triton X-100, washed with Hank's BSS 1x buffer (PAA laboratories GmbH); with or without subsequent treatement with RNase A for 7 min with (Roche Applied Science, 1 mg/ml). Cells were fixed with Dithiobis (succinimidyl propionate) (DSP) (Thermo Scientific) according to the manufacturer. Briefly, immediately before fixation DSP was added to a final concentration of 0.5 mM in 100 mM Hepes (pH 7.4) in Hank's buffer. Cells were fixed in the freshly prepared DSP solution for 90 minutes and then incubated for 30 minutes in quenching solution (50 mM monoethanolamin, 0.1% Triton X100 in Hank's buffer). Immunostaining was performed with anti-SC35 (Novus Biologicals) and anti-p68 (Santa Cruz Biotechnology) antibodies; immunofluorescent images were recorded on a Nikon confocal microscope. For statistical analysis, the nuclear pattern of EGFP-Gadd45a was assessed manually (n = 35–50).

For detection of endogenous Gadd45a HEK293T cells grown on coverslips and subjected to UV irradiation (40 mJ/cm2) were permeabilized with 0.2% Triton X-100 in 20 mM Tris-HCl (pH 7.4), 5 mM MgCl_2_, 0.5 mM EDTA, and 25% glycerol; with or without subsequent RNase treatment as described above. Cells were fixed with 2% formaldehyde and antigen retrieval was performed as described [34], except that microwave treatment was done at 450W. Immunofluorescence was performed using anti-Gadd45a H165 antibody (Santa Cruz). Statistical analysis of the endogenous Gadd45 nuclear patterns was performed manually (n = 50).

### Nuclear extract preparation

HEK293T or RKO cells were harvested, washed twice with DPBS buffer, and homogenized in buffer A (0.3 M sucrose, 10 mM Tris-HCl pH 8.0, 3 mM CaCl_2_, 2.5 mM Magnesium acetate, 0.25% Triton X-100, 0.1 mM EDTA, 2 mM DTT, Complete™ proteinase inhibitors (Roche)) in a douncer homogeniser. Homogenates were mixed 1∶1 with buffer B (1.8 M sucrose, 10 mM Tris-HCl pH 8.0, 3 mM CaCl_2_, 2.5 mM magnesium acetate, 0.1% Triton X-100, 0.3 mM EDTA, 2 mM DTT, CompleteTM proteinase inhibitors (Roche)), and centrifuged through a buffer B cushion for 20 min at 13,000 g in a Beckman SW41 Ti rotor. The pellet was resuspended in nuclear extraction buffer (50 mM Hepes-NaOH (pH 7.8), 140 mM NaCl, 1 mM MgCl_2_, 1 mM DTT, 0.1 mM Vanadyl ribonucleosides (Sigma), proteinase inhibitors (Roche) and sonicated. Nuclear extract was centrifuged at 15000 g for 30 min, and the supernatant was used for further procedures.

### Sucrose gradient sedimentation analysis

Sucrose gradient sedimentation analysis was performed using nuclear extract from 2×10^7^ RKO cells. Samples were untreated or treated with 100 µg/ml of ribonuclease A (Roche) or 40 U/ml of DNase I (MBI) for 30 min at room temperature. Soluble nuclear proteins were applied to the top of a 8–40% sucrose gradient and centrifuged for 26 h at 50000 g at 4°C. Samples containing sedimentation markers thyroglobulin (19S), β-galactosidase (16.4 S), catalase (11S) or a cytoplasmic fraction containing ribosomal subunits were run separately. Proteins from gradient fractions were precipitated and analyzed on immunoblots.

### Filter binding assay

Filter binding assays were performed essentially as described [35]. Recombinant proteins used were bovine serum albumin (Fraction V, Sigma), His-Gadd45a and M-MLV-reverse transcriptase (Invitrogen). Binding reactions were performed in RNA binding buffer (10 mM Tris-HCl (pH 7.4), 10 mM KCl, 1 mM MgCl_2_). In reactions without competitor, 2.5 or 1.3 µM recombinant proteins were preincubated for 20 min with 10,000 cpm (approximately 2 ng) of ^32^P-labelled multiple cloning site (MCS) RNA of pCS2 and pXT1 plasmids. In competition assays, unlabeled competitor nucleic acids were preincubated for 10 min with recombinant proteins before addition of labeled RNA. Reactions were applied to nitrocellulose filters that were pre-blocked with 50 µg/ml BSA in RNA binding buffer, washed with RNA binding buffer and quantified by scintillation counting.

### Structural data files

Apart from the crystal structures used as templates for xtGadd45a homology modelling, the crystal structures of human spliceosomal p15.5 kDa protein bound to a U4 snRNA fragment (PDB ID 1e7k, [19]), yeast L30e-mRNA complex (PDB ID 1t0k, [36]), *Haloarcula marismortui* ribosomal protein L7Ae-rRNA complex (PDB ID 1s72, [37]), yeast spliceosomal protein Snu13p dimer complex (PDB ID 1zwz, [38]) were used. For homology modeling the sequences of xtGadd45a (NCBI AN CAJ82672) and human SPB2 (NCBI AN NP_076982) were used.

### Homology modeling and structure analysis

Two close homologues of xtGadd45a were used as templates for homology modeling: human (PDB ID 2wal, citation pending), and mouse (PDB ID 3cg6, [39]) Gadd45g. Initial sequence alignments were generated in ClustalX2 [40] and manually refined from 3D alignments of available crystal structures for hsp15.5, scL30e, hmL7Ae, scSnu13p, hsGadd45g and generated models of hsSBP2_RBD and xtGadd45a. Modeling of xtGadd45a was carried out as described [41], [42] using the MODELLER package [43]. In the same way a model of the xtGadd45a mutant G39A was generated. For modeling of the human SPB2 RNA-binding domain the crystal structure of yeast spliceosomal protein Snu13p (PDB ID 1zwz; [38]) was used. Initial models were scored for energy content and sterical correctness and the best model further optimized using GROMACS molecular dynamics simulations [44] was used. All models were scored for energy and sterical correctness using the ANOLEA [45], VERIFY_3D and ERRAT (http://nihserver.mbi.ucla.edu) online servers. Structure analysis was carried out using SwissPBD Viewer [46] and PyMol (www.pymol.org). PDB2PQR [47], PropKa [48] and APBS [49] packages were used for charge surface calculations and the HotPatch web server [50] for hydrophobicity calculations. For protein-protein dockings the GRAMM package was used in hydrophobic mode [51].

### Purification of recombinant his-xtGadd45a

pET28a vectors containing ORF of xtGadd45a and mutants were transformed into BL21DE3 *E.coli*. Bacteria were grown in 100 ml medium and induction was performed by IPTG for 4 h. Disruption was performed by French Press, and the lysate was cleared by centrifugation at 110.000 g for 30 min. Purification was performed by metal affinity chtomatography under native conditions on a Ni-NTA column (Qiagen) according to the manufacturer's instruction except that lysis buffer contained 1 mM of MgCl_2_ and 0.04% of NP40. Step elution with 60–100 mM imidazole was performed. Fractions were analyzed by SDS-PAGE with Coomassie staining (Thermo Scientific), and fractions eluting at 100 mM imidazole were dialyzed against lysis buffer and used for further experiments.

### Luciferase reporter assay

Dual-Luciferase reporter assays (Promega) were performed as described in [13].

### Southern blot methylation analysis

HEK293T cells were transiently transfected in 10 cm dishes with 5 µg HpaII *in vitro* methylated pOctTK-EGFP and 1.2 µg pBl-KS or xtGadd45a. Transfected plasmid DNA was recovered 72 h after transfection, digested with NotI and either HpaII or MspI and analyzed by Southern blot using a *GFP* probe. The expression of EGFP was additionally analyzed by SDS-PAGE and Western blot using anti-GFP antibody.

## Results

### Gadd45a binds RNA *in vitro* and *in vivo*

**Figure 1:**
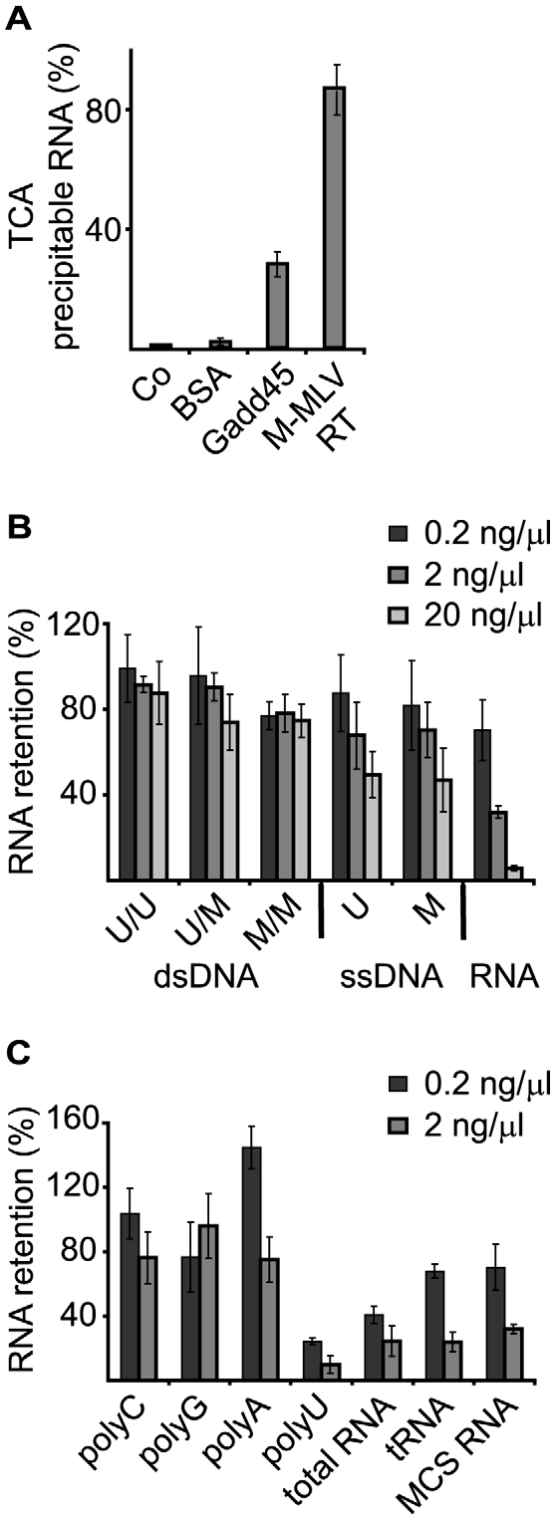
Gadd45a binds RNA *in vitro*. A, RNA filter binding assay using the indicated proteins and ^32^P-labeled RNA (multiple cloning site transcript, MCS). Co, no protein; BSA, bovine serum albumin; M-MLV RT - Moloney murine leukemia virus reverse transcriptase. B, C, RNA filter binding assays using ^32^P-labeled MCS RNA were performed with recombinant Gadd45a in the presence of the indicated unlabeled competitor nucleic acids. Data are shown as percentage of ^32^P bound in the absence of the competitor. Each sample was done in triplicate; average and standard deviation was generated; A representative experiment out of three is shown. U, unmethylated; M, methylated; U/U, unmethylated; U/M, hemimethylated; M/M, holomethylated; PolyA, polyC, polyG, polyU, homopolyribonucleotides; total RNA, RNA isolated from HEK293T cells; tRNA, yeast tRNA; MCS RNA, multiple cloning site RNA. Error bars, s.e.m. (n = 3). A representative experiment out of three is shown.

To test whether Gadd45a binds RNA, we carried out filter binding assays using recombinant Gadd45a and radiolabeled synthetic vector RNA, which indicated significant RNA binding compared with M-MLV reverse transcriptase (Figure 1A). To further characterize nucleic acid binding, filter binding assays were performed by preloading Gadd45a with unlabeled nucleic acids followed by competition with labeled RNA from a plasmid multiple cloning site (Figure 1B, C). Since Gadd45a is implicated in DNA demethylation, we tested methylated as well as unmethylated DNAs. Neither unmethylated, nor methylated single- nor double stranded DNA efficiently competed for RNA binding. Poly-uridine was the best competitor among RNA homopolymers (Figure 1C). Other complex RNAs, including tRNA, vector derived RNA, and notably total cellular RNA, were also effective.

**Figure 2:**
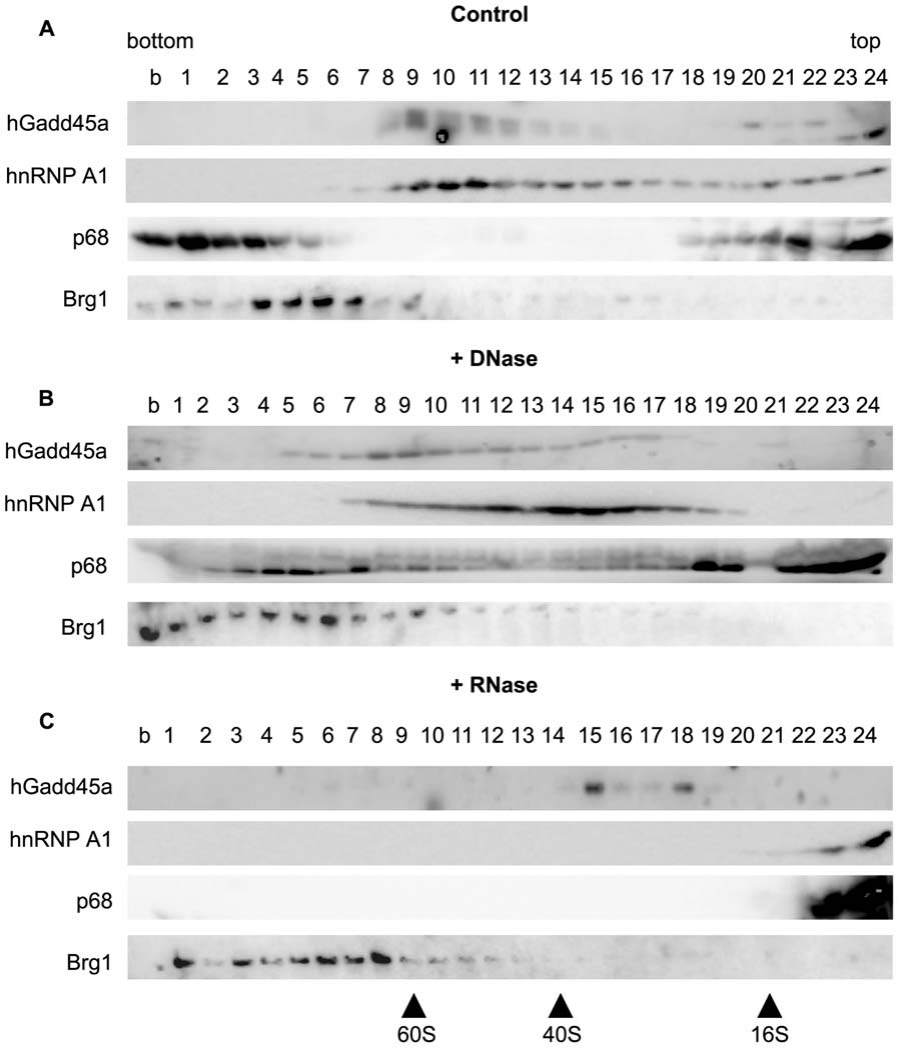
Gadd45a is part of a large RNase sensitive complex. A–C Sedimentation analysis of RKO nuclear extracts in a linear 8–40% (top-bottom) sucrose gradient. Fractions were analyzed by Western blot for Gadd45a, hnRNP A1 and p68 (control RNA binding proteins) and Brg1 (negative control). Prior to sedimentation nuclear extracts were left untreated (A), DNAse treated (B), or RNAse treated (C). b, resuspended micro-pellet of tube. Representative experiment out of three is shown.

To examine if Gadd45a is bound to RNA *in vivo*, we analyzed its sedimentation behaviour in sucrose density gradients (Figure 2). As a source we used RKO cells, which express Gadd45a endogenously [52]. Interestingly, the majority of Gadd45a sedimented in the ribosome-sized fractions, with S-values between 40 and 60 (Figure 2A). Significantly, RNase treatment shifted Gadd45a to lighter fractions, suggesting that Gadd45a may be present in an RNP-like particle (Figure 2C).

Two RNA binding proteins, ribonucleoprotein hnRNP A1, a component of ribonucleoprotein (RNP) particles [53] and RNA helicase p68 [54], were analyzed as positive controls. The ATPase Brg1 served as negative control protein. It is part of a nucleosome remodelling complex and not thought to bind RNA [55]. The sedimentation profile of hnRNP A1 was broad, with a peak in the ribosomal fractions, like for Gadd45a (Figure 2A) and consistent with it being part of RNP particles. In contrast, p68 showed a bimodal distribution, fractionating as a very heavy and a light form, as reported previously [54]. Brg1 was recovered only in heavy fractions. DNase pretreatment showed minor alterations in the sedimentation behavior of the proteins but these were within the margins of sample variability (Figure 2B). In contrast, RNAse pretreatment led to a reproducible shift in sedimentation of both RNA binding proteins hnRNPA1 and p68 to the light fraction, while it did not affect Brg1 (Figure 2C), indicating that the effect was indeed due to RNase and not contaminating protease.

The RNase sensitive sedimentaion profile of Gadd45a supports it being associated with RNA endogenously.

### Gadd45a localizes in nuclear speckles in an RNase sensitive manner

**Figure 3:**
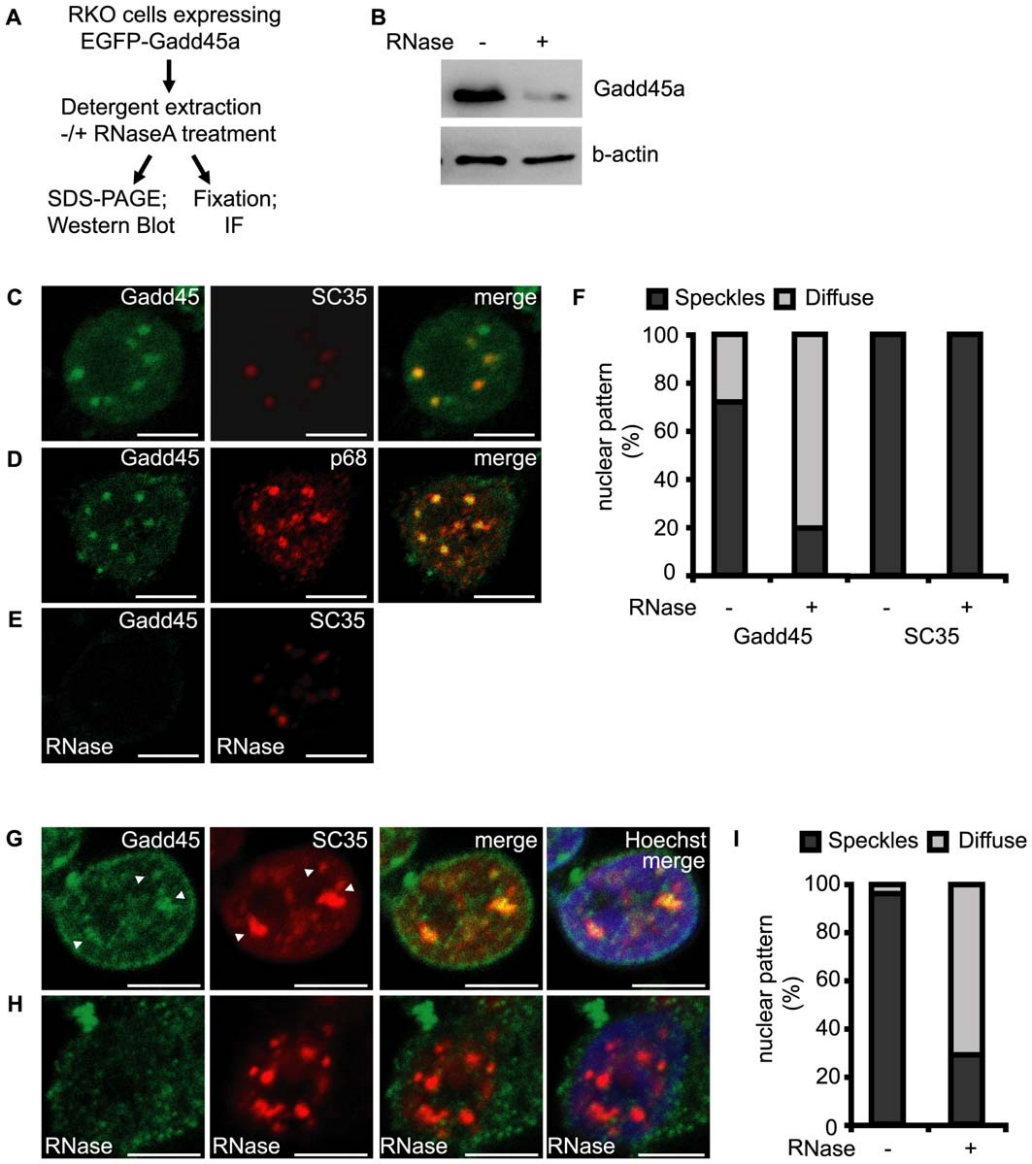
Gadd45a localization in nuclear speckles is RNase sensitive. A, Scheme of detergent extraction. B, RKO cells expressing EGFP-Gadd45a were subjected to detergent extraction with or without RNaseA treatment followed be Western blot analysis of the indicated proteins. A representative experiment out of three performed is shown. C, D Immunofluorescence confocal microscopy of detergent-extracted RKO cells. Cells were transfected with N-EGFP-Gadd45a and stained with antibodies against SC35 (C) and p68 (D). E, cells were treated as in B, but subjected to RNase treatment after extraction and before fixation. F, Statistical analysis for localization of EGFP-Gadd45a and SC-35 in nuclear speckles in cells with and without RNaseA treatment (n = 35 cells; n = 3 experiments; a representative experiment is shown). G, H Immunofluorescence confocal microscopy of endogenous Gadd45a in UV irradiated detergent-extracted HEK293T cells. Cells were stained with antibodies against Gadd45a and SC35. In (H), cells were subjected to RNase treatment after extraction and before fixation. I, Statistical analysis for localization of UV inducible Gadd45a in nuclear speckles in HEK293T cells with and without RNaseA treatment (n = 50 cells).

To further examine whether Gadd45a is a RNA binding protein we analyzed the RNase sensitivity of its localization. Cells were transfected with Gadd45a and soluble proteins were detergent extracted with or without RNase treatment and analyzed by Western blot or immunofluorescence (IF) microscopy (Figure 3A–F). In Western blot analysis Gadd45a is removed from the detergent-resistant fraction upon RNase treatment (Figure 3B). IF analysis of detergent-extracted RKO cells showed that EGFP-Gadd45a is localized in nuclear speckles, as described previously [56]. There it was colocalized with the nuclear speckle markers SC35 (Figure 3C) and p68 (Figure 3D). Nuclear speckles are the main repository for factors involved in transcription elongation, mRNA processing and export [57]–[59]. In some cells overexpressed Gadd45a strongly localized to the nuclear periphery (Figure S1). We tested if this localization is RNase sensitive. Indeed, RNase treatment reduced the number of cells where Gadd45a localized in nuclear speckles from 72% to 20% (Figure 3E, F). In contrast, RNase treatment did not affect localization of Gadd45a in the nuclear periphery (not shown) as well as SC35 staining.

In HEK293T cells endogenous Gadd45a showed only a weak, homogeneous nuclear signal. However, following UV-irradiation, which induces Gadd45a expression, the protein was colocalized with SC35, but was also found in SC35 negative punctae and in the nuclear periphery (Figure 3G). Once again, RNaseA treatment removed Gadd45a from nuclear speckles (Figure 3H, I).

The nuclear speckle co-localization with RNP proteins SC35 and p68 and its RNase sensitivity support that Gadd45a is an RNA binding protein and may be part of an RNP.

### Modeling of Gadd45a-RNA binding

To gain insight into the structural basis of Gadd45a-RNA interactions we inspected the three dimensional structures of Gadd45 as well as of structures of other L7Ae members, which were solved in complex with RNA.

**Figure 4:**
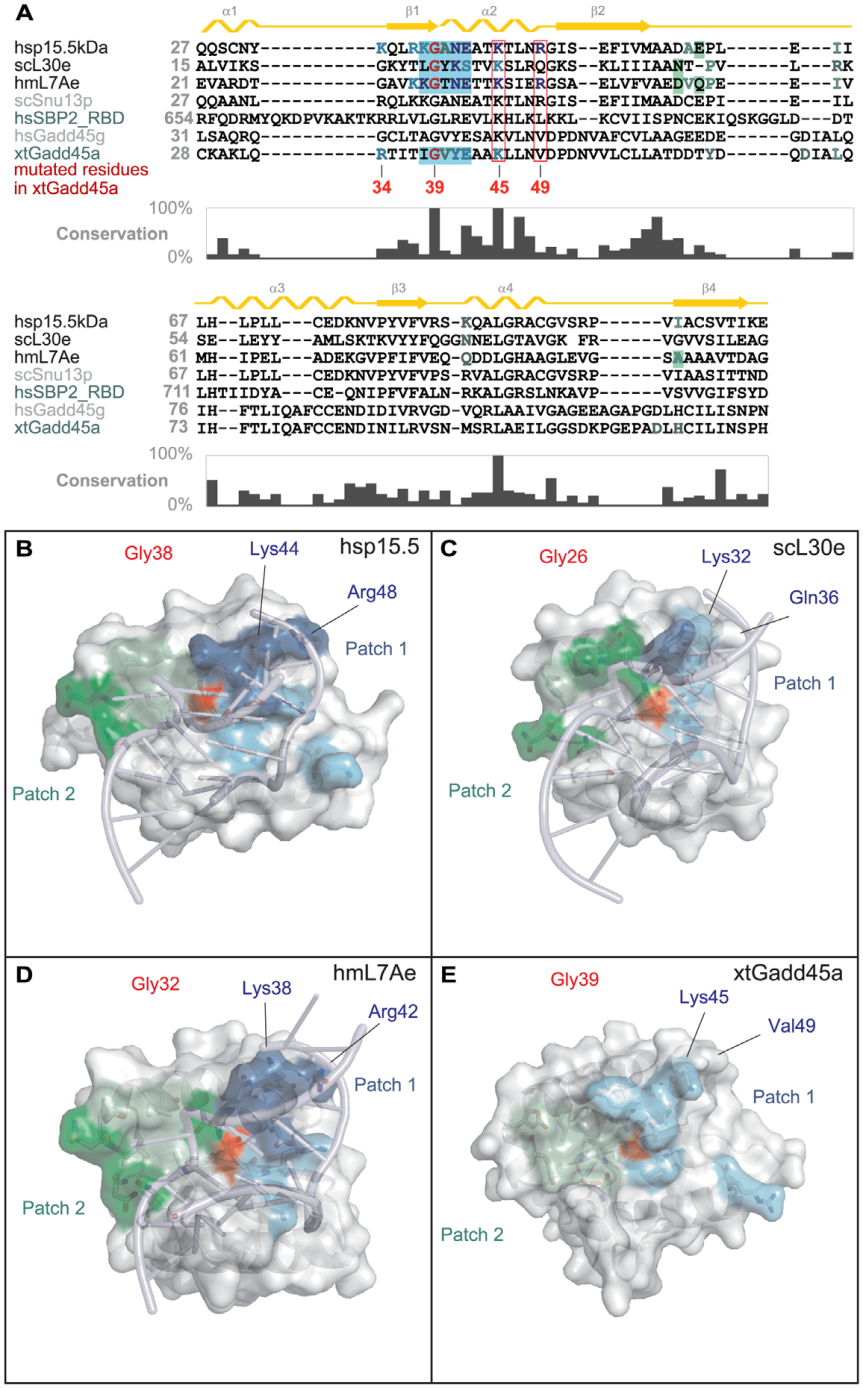
Gadd45a modeling suggests domains of RNA binding. A, Sequence alignment of L7Ae family proteins: human hsp15.5 kDa protein, yeast ribosomal scL30e protein, *Haloarcula marismortui* ribosomal hmL7Ae protein, yeast spliceosomal protein scSnu13p protein, human hsSBP2_RBD (RNA-binding domain), human hsGadd45g and *Xenopus tropicalis* xtGadd45a, including secondary structure elements (above) and sequence conservation (below). Light and dark blue letters indicate backbone- and side chain RNA interacting residues from patch 1. Light and dark green letters indicate backbone and hydrophobic side RNA interacting residues from patch 2 (see text for details). Residues targeted by mutagenesis are marked. B–E, Comparison of the crystal structures of human hsp15.5 kDa protein (B), yeast ribosomal scL30e protein (C) and *Haloarcula marismortui* ribosomal hmL7Ae protein (D), and the homology model of *Xenopus tropicalis* xtGadd45a (E). Residue coloring as above. The red area denotes the ultra-conserved Gly residue (RNA guanine G-binding region) important for specific RNA binding and DNA demethylation.

First, we built a homology model for *Xenopus tropicalis* Gadd45a by employing the available crystal structures of Gadd45g [39]. Next we compared the sequences (Figure 4A) and structures (Figure 4B–E) of the three L7Ae protein family members: human spliceosomal p15.5 kDa protein bound to a U4 snRNA fragment, yeast L30e-mRNA complex, and *Haloarcula marismortui* ribosomal protein L7Ae-rRNA complex. All three L7Ae family proteins are bound to the so-called kink-turn RNA motif. Analyzing general rules of recognition of this type of RNA, we identified two main patches on the RNA-binding surface of these proteins. In patch 1 (shades of blue in Figure 4) RNA-protein contacts are formed by positively charged amino acids from β-strand β1, helix α2 and several highly conserved amino acids. Patch 2 (green in Figure 4) represents a hydrophobic pocket able to accommodate a purine or pyrimidine base flipped from the kink-turn RNA. This pocket is formed by amino acids from different parts of the protein. Both of these patches are present in all L7Ae family member-RNA complexes. Patch 1 is relatively conserved in sequence and structure (Figure 4). Patch 2 varies in size and amino acid composition but is generally composed of hydrophobic core residues surrounded by polar amino acids. We propose that patch 1 plays a role in general protein-RNA interaction and patch 2 may be responsible for sensing the kink-turn RNA motif. Interestingly, the same conserved RNA-binding like patches are present on the surface of *Xenopus* Gadd45a (Figure 4E).

Besides the two patches, the glycine residue homologous to G39 in Gadd45a (Figure 4A; red in Figure 4B–E) is highly conserved in all L7Ae family members and constitutes a third important structural determinant for kink-turn RNA binding. This residue is located at the beginning of helix α2, further referred to as guanine (G)-binding region, since in all L7Ae-RNA structures a guanine base is tightly bound in this region through an extensive hydrogen-bonding network (Figures S2 and S3). A glycine to alanine or lysine mutation of this residue completely abrogates RNA binding and protein function in human SBP2 and p15.5 kDa protein, respectively [60], [61]. Indeed, modeling the G38A mutant of hsp15.5, we discovered that a G38A mutation should result in sterical clashes with bound RNA (Figure S3).

Taken together, our modeling and structural analysis suggests a rationale for the ability of Gadd45a to bind RNA despite its acidic pKa and the absence of a distinctly positively charged region.

### Mutations affecting Gadd45a RNA binding and demethylation

**Figure 5:**
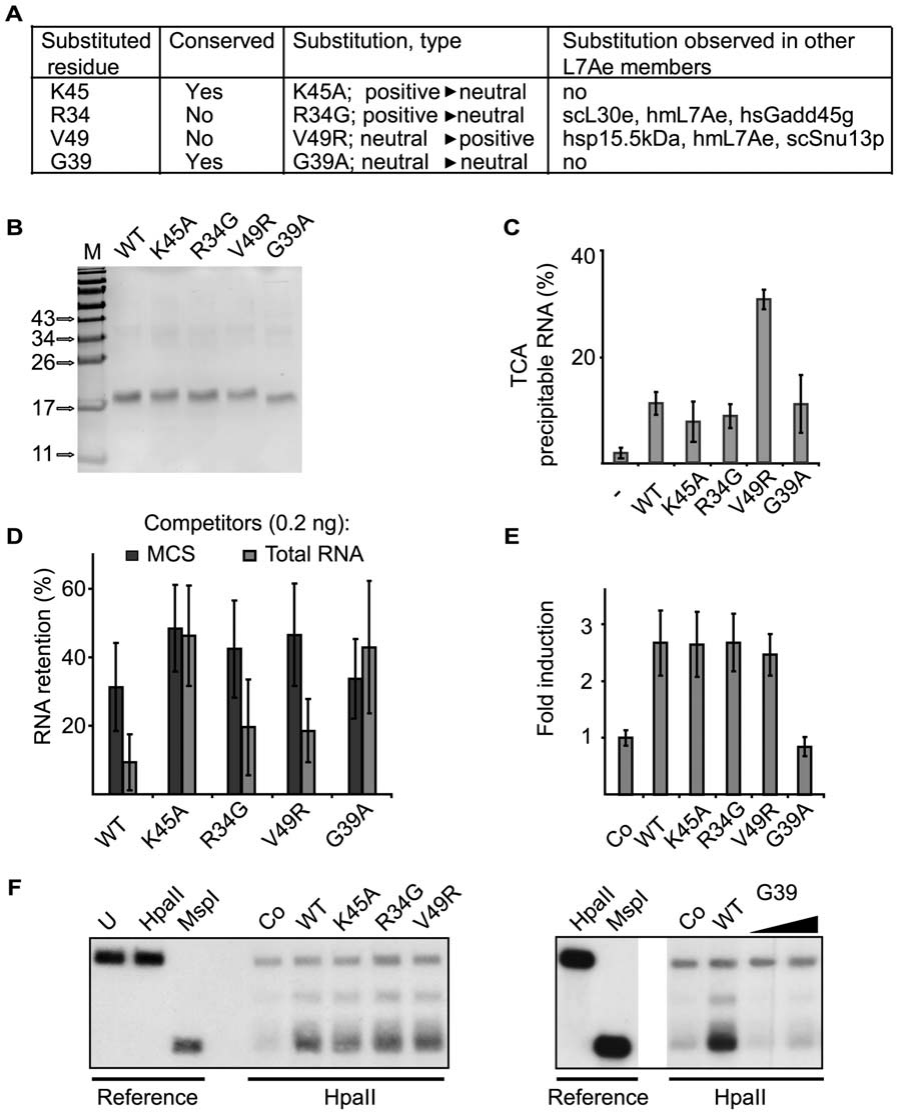
RNA binding and DNA demethylation in Gadd45a point mutants. A, general characteristics of Gadd45a point substitutions. B, SDS-PAGE analysis of His-tagged Gadd45a wild type and point mutant proteins produced and purified from *E.coli.* C, filter binding assay of Gadd45a wild type and point mutant proteins using multiple cloning site (MCS) ^32^P-RNA. D, RNA filter binding assays using ^32^P-labeled MCS RNA were performed with wild type or point mutant Gadd45a proteins in the presence of the indicated unlabeled competitor RNAs. Data are pooled from seven independent experiments. E, F DNA demethylation assays. E, Luciferase reporter assays of HEK293T cells transiently transfected with an M. SssI *in vitro* methylated SV40-luciferase reporter and the indicated constructs. Error bars, s.e.m. (n = 3). F, Methylation sensitive Southern blot of HpaII *in vitro* methylated pOctTK reporter recovered from HEK293T cells cotransfected with *Xenopus* Gadd45a wild type and mutants.

To test the role of amino acid residues in patch 1 and of G39 in RNA binding and DNA demethylation, we generated four point mutations in Gadd45a, K45A, R34G, V49R and G39A (Figure 5A). RNA binding activity of purified recombinant proteins (Figure 5B) was tested by filter binding assay with radioactively labeled vector derived RNA. Nonspecific RNA binding ability was comparable for the three mutants K45A, R34G and G39A and wild type Gadd45a (Figure 5C). The V49R substitution showed three-fold higher RNA binding ability. This may reflect increased ionic interaction upon addition of an extra positive charge in this position.

We next tested specific RNA binding by the RNA competition assay described in Figure 1C. For each mutant we compared the binding competition of labeld synthetic (vector) RNA with unlabeled cellular RNA. Since total RNA was a good competitor in the *in vitro* binding assays (Figure 1C), we reasoned that it contains relevant- but unknown RNAs, which physiologically bind to Gadd45a with high affinity. Wild type Gadd45a showed a three-fold difference in competition assays between vector- and total RNA (Figure 5D). This was similar for the R34G and V49R mutants, which harbor patch 1 substitutions naturally occurring in other L7Ae members. In contrast, substitution of either of the two ultra-conserved amino acids – K45A and G39A - caused a loss of discrimination between vector- and total RNA binding.

To test the activity of the mutants in DNA demethylation, we monitored Gadd45a-mediated re-activation of an *in vitro* methylated – and hence silenced - luciferase reporter plasmid. Gadd45a can demethylate and thus transcriptionally activate such reporters [13]. Upon transfection in HEK293T cells wild type Gadd45a as well as R34G and V49R mutants equally activated the methylated reporter (Figure 5E). Notably the K45A mutant, which failed to discriminate between specific and non-specific RNA binding, was fully active in the demethylation assay, indicating that the two properties can be uncoupled. This already suggests that specific RNA binding is not absolutely essential for DNA demethylation, at least under these experimental conditions. In contrast, Gadd45a G39A was the only mutant inactive in the reporter assay as well as in specific RNA binding.

To test for gene specific demethylation, we transfected a methylated EGFP expression plasmid and monitored its methylation status by digestion with the methylation sensitive endonuclease HpaII. In parallel we measured its expression by detecting EGFP protein in the cell lysates. The analysis showed that cotransfection of wild type, K45A, R34G and V49R led to expression of EGFP protein and to the appearance of a HpaII cleavage product, indicative of demethylation (Figure S4 and Figure 5F). The G39A mutant, which failed to discriminate between specific and non-specific RNA binding, was also inactive in DNA demethylation as well as activation of EGFP expression.

**Figure 6:**
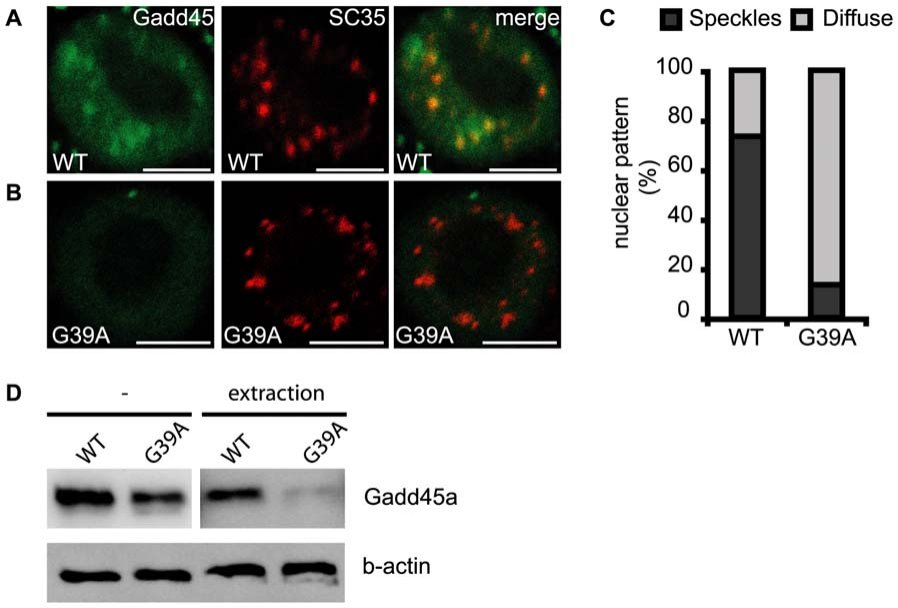
G39A substitution weakens Gadd45a association with nuclear speckles. A,B IF microscopy comparison of nuclear pattern after detergent extraction of EGFP-Gadd45a wild type (A) and EGFP-G39A mutant (B). Experiments were done essentially as in Figure 3A. C, Statistical analysis of immunofluorescence patterns as in Figure 3F. D, Western blot analysis of RKO cells expressing EGFP-Gadd45a wild type (wt) or EGFP-G39A mutant harvested without or after detergent extraction. Scale bar, 4 µm.

Finally, we tested, if the G39A mutant protein still localizes to nuclear speckles. Nuclear localization of the G39A mutant was much reduced in general and less than 20% showed nuclear speckles (Figure 6A-C). Similarly, Western blot analysis of detergent treated cells showed that G39A was more sensitive to extraction than wild type Gadd45a (Figure 6D).

From the mutant analysis we conclude that a) specific RNA binding is not absolutely essential for DNA demethylation and b) that G39 is a critical amino acid for the function and localization of Gadd45a.

## Discussion

The main findings of this study are that Gadd45a is an RNA binding protein and that it appears to be part of an RNP particle. This is in line with the function of other members of the L7Ae/L30e/S12e superfamily, which are either ribosomal components or associated with RNP particles. Gel filtration and cross linking analysis of recombinant Gadd45b,g indicates that the protein forms a dimer of 35 kDa [39], [62], while Gadd45a can oligomerize [3], suggesting that the cellular high molecular weight form of Gadd45a may contain multimers.

The conclusion that Gadd45a is in an RNP complex is supported by sucrose density gradient centrifugation and its localization in nuclear speckles. It is interesting that nuclear speckles are a site of active transcription, RNA splicing and processing. This raises the possibility that Gadd45a RNPs are associated with genes undergoing active DNA demethylation and transcriptional activation. Of note, p68/Ddx5, which colocalizes with Gadd45a in nuclear speckles, was previously described as a component of a DNA demethylase complex [26], [27].

RNP complexes play prominent roles in RNA processing, RNA transport and RNA translation (for review, see [63]–[65]). In light of our results it is interesting that overexpression of Gadd45 leads to a similar phenotype in *Drosophila* as mutation of *squid*, which encodes an hnRNP. In both cases the chorion of fly eggs is dorsalized due to defects in *grk* mRNA localization and translation [66]–[68], supporting the idea that a Gadd45 RNP function is evolutionary conserved.

Our *in silico* modeling suggests a structural basis for the RNA binding of Gadd45a. Like other RNA binding proteins of the L7Ae family, Gadd45a contains two patches, which appear to be involved in RNA binding. The *in vitro* RNA binding assays and point mutagenesis data suggest that Gadd45a has moderate affinity for nonspecific RNAs and high affinity for specific RNAs. The V49R substitution increased non-specific RNA binding. Since arginine instead of valine 49 is a naturally occurring variant in some L7Ae superfamily proteins, we propose that such proteins have a higher general RNA binding propensity.

The specific RNA binding of Gadd45a is clearly not absolutely essential for its demethylating activity, as shown by the K45A mutant. However, our DNA demethylation assay chosen for convenience is rather artificial; it involves an abundant *in vitro* methylated reporter plasmid, which is demethylated by overexpressed Gadd45a. In contrast, locus-specific demethylation under physiological conditions may very well require its ability to bind specific RNAs as discussed below.

The G39A substitution in the G-binding region abolished both DNA demethylation as well as specific RNA binding ability. Since the K45A mutant still demethylates despite inactivated specific RNA binding, the RNA binding defect of G39A may not be the cause for the demethylation defect. This raises the possibility that G39A interferes with some other important property of Gadd45a. However, at least dimerisation of Gadd45a does not seem to be affected by G39A since by *in silico* docking of Gadd45a dimers [39], G39 was not found in the vicinity of the putative Gadd45a dimer interface (Figure S5).

Our study raises new questions concerning the biology and biochemistry of Gadd45 proteins. Which RNAs are physiologically bound to Gadd45? What other proteins are parts of the Gadd45 RNP particle? Is the role of Gadd45 bound RNAs purely structural or is RNA involved in e.g. specific targeting to demethylated DNA regions?

## Supporting Information

Figure S1 Perinuclear pattern of Gadd45a. Immunofluorescence confocal microscopy of detergent-extracted RKO cells. Cells were transfected with EGFP-xtGadd45a and developed with antibody against SC35; nuclei were stained with Hoechst. This pattern is observed in ∼10% of EGFP-Gadd45 positive cells. Scale bar, 5 µm.
(1.05 MB JPG)

Figure S2 Possible H-bonding networks in patch 2 and G-binding region in human hsp15.5 kDa protein (A), yeast ribosomal scL30e protein (B), Haloarcula marismortui ribosomal hmL7Ae protein (C) and the model of xtGadd45a (D). Residues colored in light and dark blue form patch 1 and those colored in light and dark green form patch 2, respectively (see also Figure 4 legend for details). The red area denotes the highly conserved Gly residue (RNA guanine G-binding region) found to be important for proper RNA binding. Left subpanels show how the flipped RNA base is sensed and accommodated in the patch 2 pocket by hydrophobic interactions of the purine or pyrimidine base with sidechains of hydrophobic residues (colored in green). The hydrogen bonding with backbone and/or sidechains of some charged amino acids surrounding the hydrophobic pocket is also shown. Right subpanels show the extensive hydrogen bonding network which could be formed by the RNA base (in all three discussed crystal structures it is always guanine). For xtGadd45a these small subpanels show modeled interactions of guanidine and uridine bases in the G-binding region and patch 2 hydrophobic pocket, respectively. RNA is shown in a semitransparent cartoon representation.
(1.92 MB JPG)

Figure S3 Modeling the Gly to Ala mutation on the basis of the hsp15.5-RNA complex. Panel A represents possible the hydrogen bonding network formed by the guanine base which is properly bound and oriented in the G-binding region. Panel B shows surface representations of the same structure illustrating that the guanine base perfectly fits into the G-binding region without any sterical clashes. Exchange of glycine residue for alanine leads would lead to considerable sterical clashes (C). To resolve these clashes we propose that the guanine base moves out from the G-binding site (D) leading to a loss of most hydrogen bonds (E).
(1.08 MB JPG)

Figure S4 Induction of EGFP expression from HpaII methylated promoter by xtGadd45a wild type and mutants. Western blot analysis of EGFP induction from HpaII methylated pOctTK-GFP reporter, as well as of xtGadd45a wild type and mutants expression. Loading was controlled using histone H3. A representative experiment out of three independent experiments is shown.
(0.49 MB JPG)

Figure S5 A, crystal structure of a yeast spliceosomal protein scSnu13p dimer complex with bound RNA molecules modeled on the hsp15.5-RNA complex crystal structure by fitting corresponding scSnu13p and hsp15.5 proteins in SwissPDB Viewer. B, model of xtGadd45a dimer complex obtained by GRAMM docking in hydrophobic mode with RNA molecules superimposed from hsp15.5-RNA complex crystal structure by fitting corresponding xtGadd45a and hsp15.5 proteins in SwissPDB Viewer. In both cases the red area on the protein surfaces represents the conserved glycine of the G-binding region. From these structures it is evident that dimerization and RNA-binding interfaces (patches 1 and 2) do not overlap.
(0.69 MB JPG)
