# Supplementary figures and images for: Gadd45a Is an RNA Binding Protein and Is Localized in Nuclear Speckles
Source: PLoS One. 2011 Jan 7;6(1):e14500. doi: 10.1371/journal.pone.0014500 (PMC3017548; doi:10.1371/journal.pone.0014500)

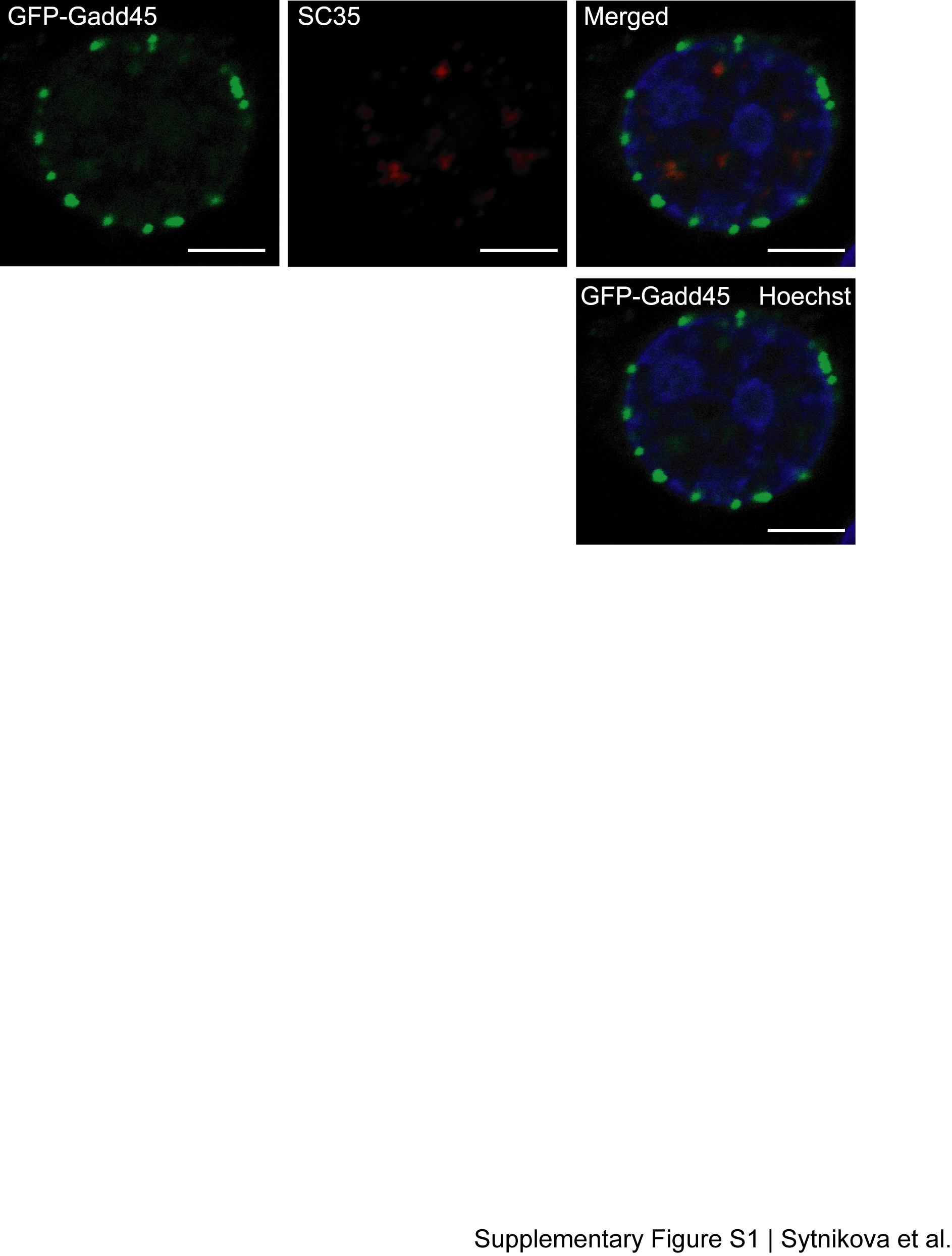

Supplement: Figure S1 — Perinuclear pattern of Gadd45a. Immunofluorescence confocal microscopy of detergent-extracted RKO cells. Cells were transfected with EGFP-xtGadd45a and developed with antibody against SC35; nuclei were stained with Hoechst. This pattern is observed in ∼10% of EGFP-Gadd45 positive cells. Scale bar, 5 µm. (1.05 MB JPG) [file pone.0014500.s001.jpg]

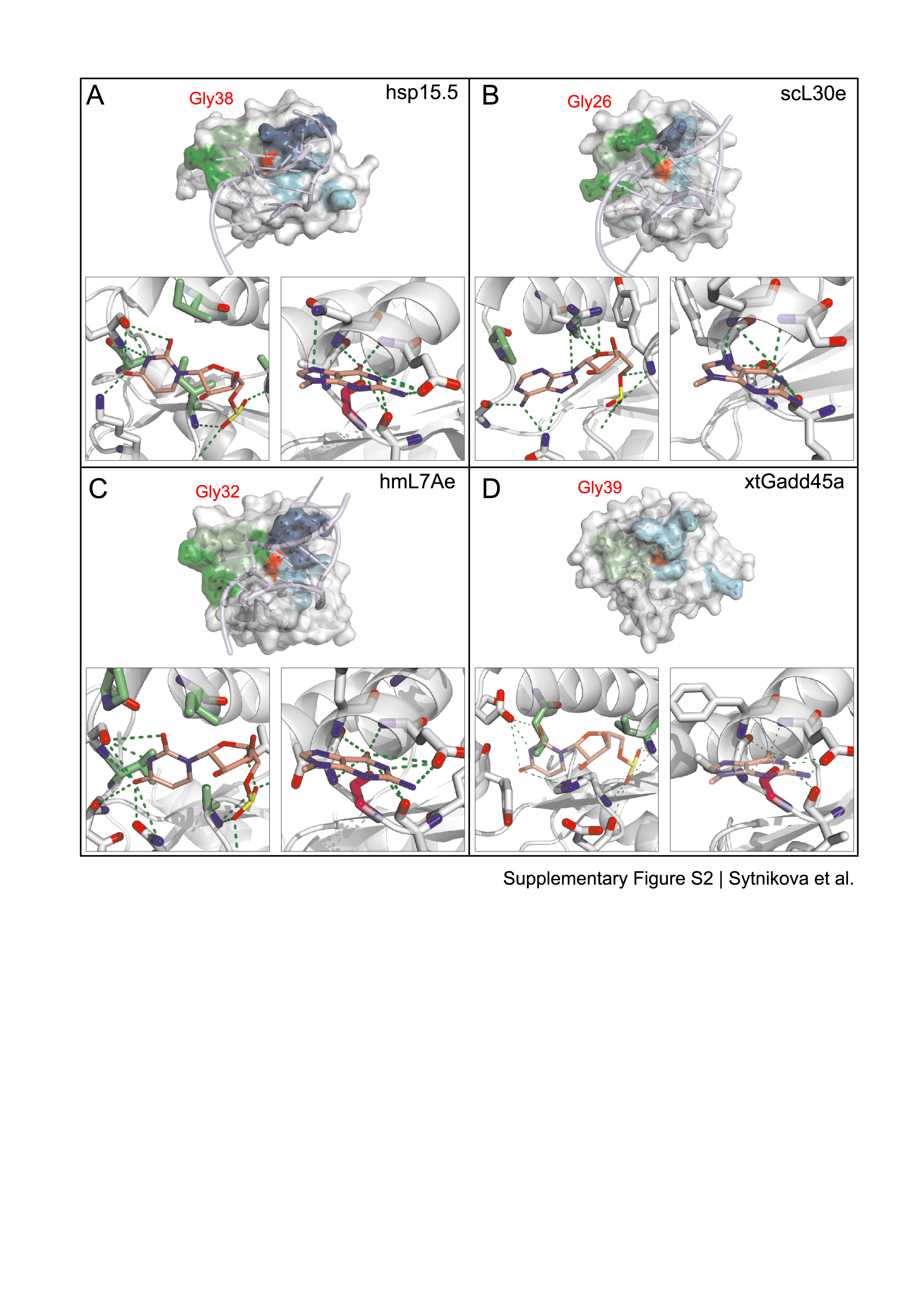

Supplement: Figure S2 — Possible H-bonding networks in patch 2 and G-binding region in human hsp15.5 kDa protein (A), yeast ribosomal scL30e protein (B), Haloarcula marismortui ribosomal hmL7Ae protein (C) and the model of xtGadd45a (D). Residues colored in light and dark blue form patch 1 and those colored in light and dark green form patch 2, respectively (see also Figure 4 legend for details). The red area denotes the highly conserved Gly residue (RNA guanine G-binding region) found to be important for proper RNA binding. Left subpanels show how the flipped RNA base is sensed and accommodated in the patch 2 pocket by hydrophobic interactions of the purine or pyrimidine base with sidechains of hydrophobic residues (colored in green). The hydrogen bonding with backbone and/or sidechains of some charged amino acids surrounding the hydrophobic pocket is also shown. Right subpanels show the extensive hydrogen bonding network which could be formed by the RNA base (in all three discussed crystal structures it is always guanine). For xtGadd45a these small subpanels show modeled interactions of guanidine and uridine bases in the G-binding region and patch 2 hydrophobic pocket, respectively. RNA is shown in a semitransparent cartoon representation. (1.92 MB JPG) [file pone.0014500.s002.jpg]

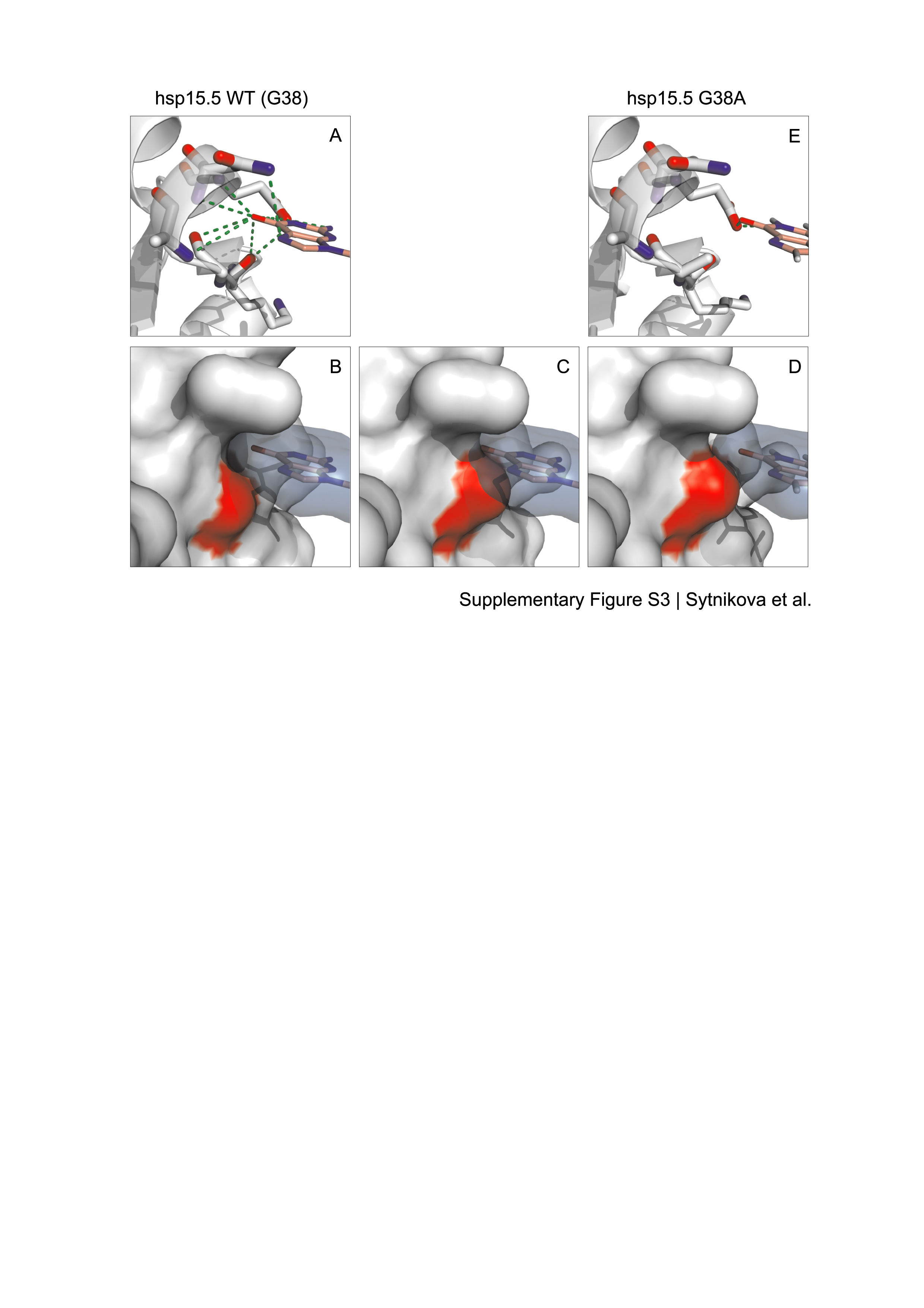

Supplement: Figure S3 — Modeling the Gly to Ala mutation on the basis of the hsp15.5-RNA complex. Panel A represents possible the hydrogen bonding network formed by the guanine base which is properly bound and oriented in the G-binding region. Panel B shows surface representations of the same structure illustrating that the guanine base perfectly fits into the G-binding region without any sterical clashes. Exchange of glycine residue for alanine leads would lead to considerable sterical clashes (C). To resolve these clashes we propose that the guanine base moves out from the G-binding site (D) leading to a loss of most hydrogen bonds (E). (1.08 MB JPG) [file pone.0014500.s003.jpg]

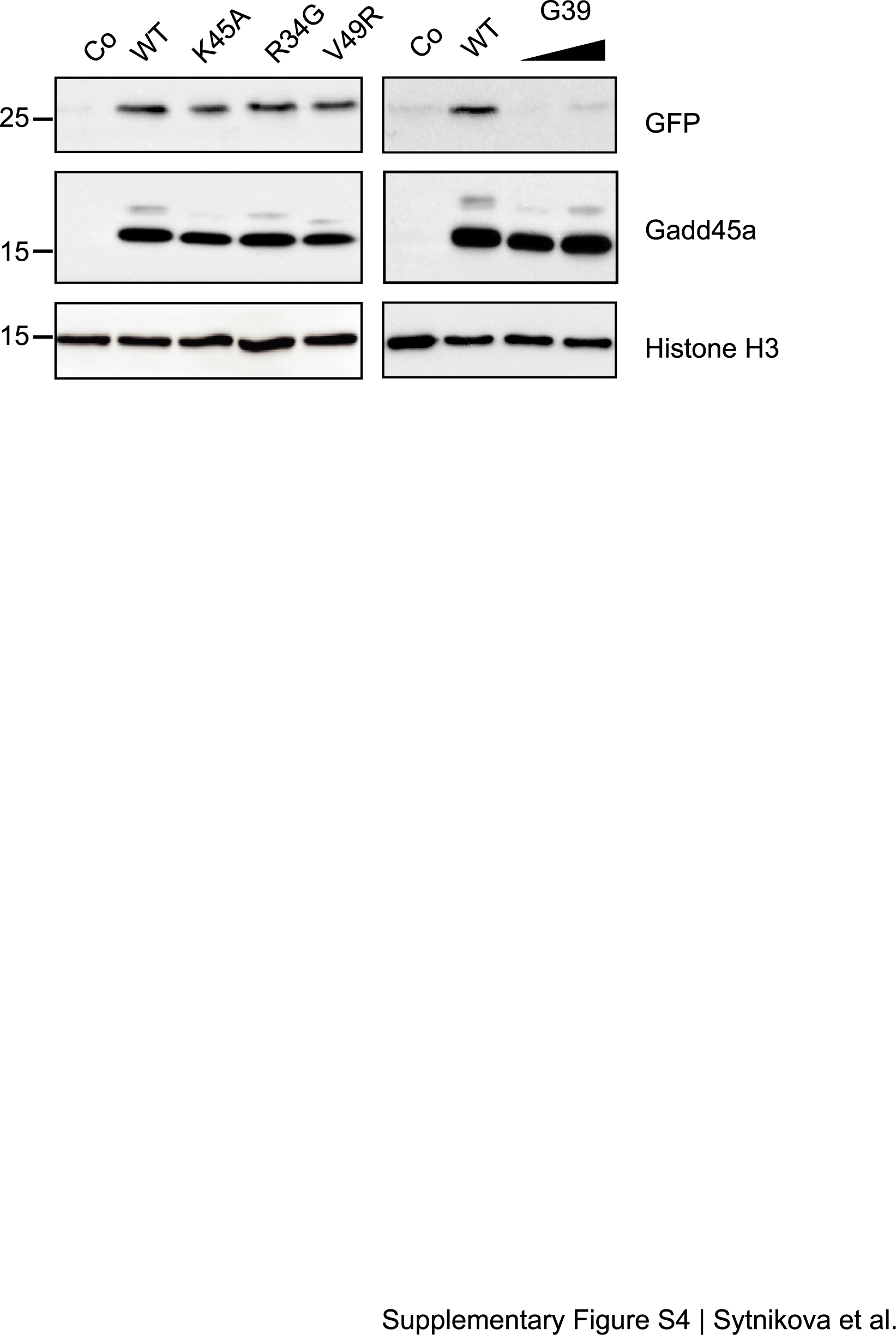

Supplement: Figure S4 — Induction of EGFP expression from HpaII methylated promoter by xtGadd45a wild type and mutants. Western blot analysis of EGFP induction from HpaII methylated pOctTK-GFP reporter, as well as of xtGadd45a wild type and mutants expression. Loading was controlled using histone H3. A representative experiment out of three independent experiments is shown. (0.49 MB JPG) [file pone.0014500.s004.jpg]

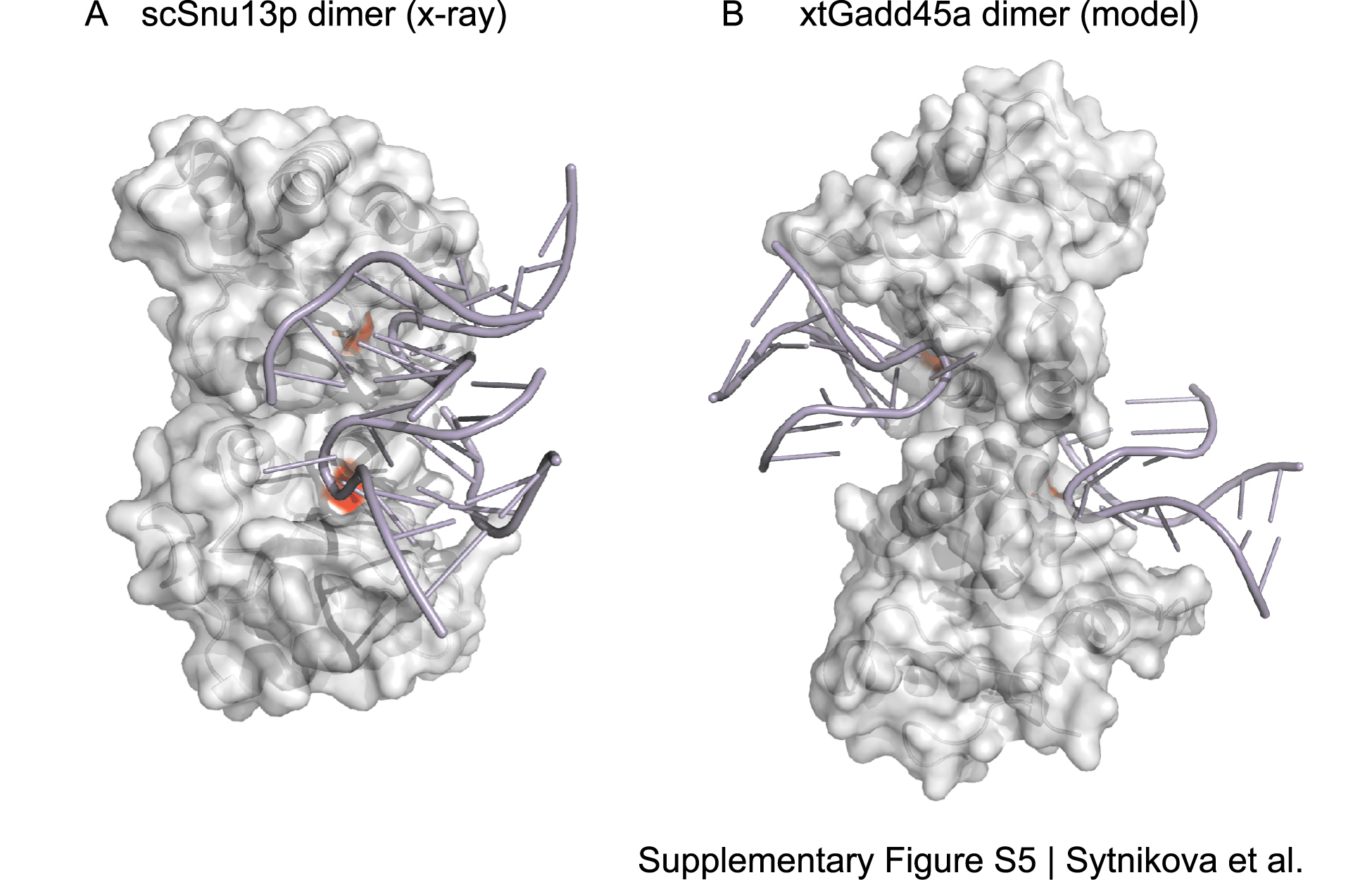

Supplement: Figure S5 — A, crystal structure of a yeast spliceosomal protein scSnu13p dimer complex with bound RNA molecules modeled on the hsp15.5-RNA complex crystal structure by fitting corresponding scSnu13p and hsp15.5 proteins in SwissPDB Viewer. B, model of xtGadd45a dimer complex obtained by GRAMM docking in hydrophobic mode with RNA molecules superimposed from hsp15.5-RNA complex crystal structure by fitting corresponding xtGadd45a and hsp15.5 proteins in SwissPDB Viewer. In both cases the red area on the protein surfaces represents the conserved glycine of the G-binding region. From these structures it is evident that dimerization and RNA-binding interfaces (patches 1 and 2) do not overlap. (0.69 MB JPG) [file pone.0014500.s005.jpg]
